# Autoantibodies against desmoglein 2 are not pathogenic in pemphigus^☆^

**DOI:** 10.1016/j.abd.2021.06.004

**Published:** 2022-01-17

**Authors:** Marcela Calixto Brandão Miguel, Tamiris Amanda Julio, Sebastian Vernal, Natália Aparecida de Paula, Andre Lieber, Ana Maria Roselino

**Affiliations:** a Division of Dermatology, Department of Internal Medicine, Faculdade de Medicina de Ribeirão Preto, Universidade de São Paulo, Ribeirão Preto, SP, Brazil; b Laboratory of Dermatology, Division of Dermatology, Department of Internal Medicine, Hospital das Clínicas, Faculdade de Medicina de Ribeirão Preto, Universidade de São Paulo, Ribeirão Preto, SP, Brazil; c Division of Medical Genetics, Department of Pathology, University of Washington, Seattle, WA, USA

**Keywords:** Acantholysis, Desmoglein 2, Epitopes, Pemphigus

## Abstract

**Background:**

Anti-desmoglein 1 and 3 autoantibodies justify acantholysis in pemphigus; however, the pathogenesis of anti-desmoglein 2 is hypothetical.

**Objective:**

To compare the participation of desmogleins 1, 2 and 3 through the production of serum autoantibodies, and protein and gene expression in the skin/mucosa of patients with pemphigus foliaceus and pemphigus vulgaris.

**Methods:**

The autoantibodies were titrated by ELISA in 202 samples of pemphigus foliaceus, 131 pemphigus vulgaris, 50 and 57 relatives of patients with pemphigus foliaceus and pemphigus vulgaris, respectively, and 114 controls. Protein and gene expressions were determined by immunohistochemistry and qPCR in the skin/mucosa of 3 patients with pemphigus foliaceus and 3 patients with pemphigus vulgaris.

**Results:**

Higher titers of anti-desmoglein 2 (optical density) resulted in pemphigus foliaceus and pemphigus vulgaris, when compared to controls (0.166; 0.180; 0.102; respectively; p < 0.0001). There was a correlation between anti-desmoglein 2 and anti-desmoglein 1 titers in pemphigus foliaceus (r = 0.1680; p = 0.0206). There was no cross-reaction of anti-desmoglein 2 with desmoglein 1 and 3. Protein overexpression of desmoglein 2 was observed in intact and lesional skin of patients with pemphigus compared to the skin of controls. Internalization granules of desmoglein 1 and 3, but not of desmoglein 2, were observed in lesions of pemphigus foliaceus and pemphigus vulgaris, respectively. Gene overexpression of desmoglein 2 was observed in the mucosa.

**Study limitations:**

Small sample size for the statistical analysis of protein and gene expression.

**Conclusion:**

Autoantibodies against desmoglein 2 are not pathogenic in pemphigus; protein and gene overexpression of desmoglein 2 in the skin and mucosa may be involved in acantholysis repair.

## Introduction

Pemphigus are autoimmune bullous diseases characterized by the production of IgG autoantibodies against desmogleins (Dsg) 1 and 3, which break the adhesion of keratinocytes, causing acantholysis. In pemphigus foliaceus (PF), the production of anti-Dsg1 is responsible for the exclusive skin involvement; in pemphigus vulgaris (PV), the production of anti-Dsg3 affects the mucosa, and when the skin is involved, anti-Dsg1 and anti-Dsg3 are detected.^1^, ^2^ Both PF and PV are prevalent in the southeast of Brazil, and the analysis of the HLA profile of patients with PF and PV confirmed that they were distinct bullous diseases.^3^, ^4^

The participation of Dsg2 is poorly studied in the pathogenesis of pemphigus. In contrast to the expressions of Dsg1 and Dsg3, which are restricted to stratified epithelia, Dsg2 is widely expressed in all epithelia containing desmosomes, including pulmonary, gastrointestinal, renal and myocardial tissues, and in the skin, ovarian, lung and stomach tumors.^5^, ^6^, ^7^, ^8^, ^9^, ^10^, ^11^ In human skin, Dsg2 is poorly expressed, being restricted to the basal layer of the epidermis, while its expression resembles the expressions of Dsg1 and Dsg3 in immortalized squamous cell carcinoma cell lines.^12^

To contribute to the understanding of Dsg2 participation in the pathogenesis of pemphigus, the production of autoantibodies against Dsg 1, 2 and 3 were compared in a large sample of patients with PF and PV, their family members, and healthy controls living in the northeast region of the state of São Paulo, Brazil, where both PF and PV are prevalent. Also, the protein and gene expressions of Dsg 1, 2 and 3 in the skin and mucosal samples obtained from patients with PF and PV were measured.

## Materials and methods

### Ethics

The Human Ethics Committee (CAAE: 56944116.1.0000.5440) approved this study in accordance with the ethical standards of the Declaration of Helsinki of the World Medical Association. All participants signed the free and informed consent form.

### Study population

Serum samples from patients with PF and PV, diagnosed by clinical, histopathology, and direct and/or indirect immunofluorescence tests; of first- and second-degree relatives and of controls (CTL), who accompanied the patients during the outpatient consultation, were obtained from 1997 to 2016. Five groups were analyzed: 202 patients with PF and 131 with PV, 50 family members of the PF group (FPF), 57 family members of the PV group (FPV), and 114 CTL. Treatment-naïve patients were classified according to their clinical form, that is, localized or generalized PF, and exclusively mucosal or mucocutaneous types of PV. Patients with PF and PV were divided into five subgroups, according to disease activity and whether or not they were undergoing treatment at the time of serum collection: treatment-naïve; with active lesions and no treatment for more than 60 days; undergoing treatment with active lesions; undergoing treatment without active lesions; and patients in remission (patients without active lesions and without immunosuppressive treatment for at least 60 days; Table 1).

**Table 1:** Demographic and clinical data, and anti-desmoglein (Dsg) ELISA results comparing patients with PF and PV, relatives of PF and PV (FPF and FPV) and Controls (CTL).

	PF	PV	FPF	FPV	Controls	p
	n = 202	n = 131	n = 50	n = 57	n = 114	
**Median age in years (25^th^/75^th^ quartiles)**	35 (21/54)^a^	48 (35/62)^b^	40 (29/49)^c^	30 (19/45)^d^	59 (45/71)^e^	0.0001^a.b^
						> 0.9999^a.c^
						< 0.0001^a.e^
						0.0001^b.d^
						0.0068^b.e^

	n (%)	n (%)	n (%)	n (%)	n (%)	
	202	131	50	57	114	
**Sex**						0.0033^a.b^
						0.0027^b.e^
Female	102 (50.2)^a^	88 (67.7)^b^	36 (72.0)^c^	40 (70.2)^d^	55 (48.2)^e^	0.8150^a.e^
						1.0000^c.d^
Male	101 (49.8)	42 (32.3)	14 (28.0)	17 (29.8)	59 (51.8)	0.0022^c.e^
						0.0088^d.e^

Disease duration (years)*	n = 199	n = 130				
< 1	45 (22.6)	31 (23.8)	NA	NA	NA	0.8967
1 to -5	87 (43.7)	86 (66.2)	NA	NA	NA	0.0296
6 to -10	32 (16.1)	9 (6.9)	NA	NA	NA	0.0390
11 to 15	11 (5.5)	3 (2.3)	NA	NA	NA	0.2630
> 15	24 (12.1)	1 (0.8)	NA	NA	NA	0.0001

Clinical form (treatment-naïve patients)*	n = 68	n = 26				
Exclusively mucosal	NA	8 (30.8)^b^	NA	NA	NA	^a^ = 0.9636
						^b^ = 0.3909
Muco-cutaneous	NA	18 (69.2)^b^	NA	NA	NA	
Localized	34 (50.0)^a^	NA	NA	NA	NA	
Generalized	34 (50.0)^a^	NA	NA	NA	NA	

Treatment on the date of serum collection*	n = 186	n = 127				
Treatment-naïve	43 (23.1)^a^	20 (15.8)^b^	NA	NA	NA	^a^< 0.0001
No treatment with active lesions	34 (18.3)^a^	11 (8.7)^b^	NA	NA	NA	^b^< 0.0001
Undergoing treatment without active lesions	16 (8.6)^a^	24 (18.8)^b^	NA	NA	NA	
Undergoing treatment with active lesions	80 (43.0)^a^	65 (51.2)^b^	NA	NA	NA	
In remission	13 (7.0)^a^	7 (5.5)^b^	NA	NA	NA	

Anti-Dsg2	n = 202	n = 131	n = 50	n = 57	n = 114	< 0.0001^a.e^
Above the cutoff	129 (63.9)^a^	87 (66.4)^b^	19 (38.0)^c^	42 (73.7)^d^	31 (27.2)^e^	< 0.0001^b.e^
						0.0012^a.c^
Below the cutoff	73 (36.1)	44 (33.6)	31 (62.0)	15 (26.3)	83 (72.8)	< 0.0001^c.d^
						< 0.0001^d.e^
**Anti-Dsg1**						< 0.0001^a.b^
						< 0.0001^a.e^
Above the cutoff	179 (88.2)^a^	67 (51.5)^b^	6 (12.0)^c^	1 (1.8)^d^	4 (3.5)^e^	< 0.0001^a.c^
						< 0.0001^b.d^
Below the cutoff	23 (11.8)	64 (48.5)	44 (88.0)	56 (98.2)	110 (96.5)	< 0.0001^b.e^
						0.0325^c.d^
**Anti-Dsg3**						
Above the cutoff	9 (4.5)^a^	102 (78.5)^b^	4 (8.0)^c^	0 (0.0)^d^	3 (2.6)^e^	< 0.0001^a.b^
						< 0.0001^b.e^
Below the cutoff	193 (95.5)	29 (21.5)	46 (92.0)	57 (100.0)	111 (97.4)	< 0.0001^b.d^
						0.0446^c.d^

NA, Not Applicable; Anti-Dsg1 and anti-Dsg3, cut-off< 20 U/mL (ELISA, MBL, Japan); Anti-Dsg2, cut-off = 0.1365 DO (in-house ELISA).

* Some data not compiled.

### Detection of IgG autoantibodies against Dsg1 and Dsg3 by ELISA

The samples were tested with a commercial ELISA kit (MBL, Nagoya, Japan) according to the manufacturer’s recommendations (cut-off = 20 U/mL).

### Detection of IgG autoantibodies against Dsg2 by in-house ELISA

Briefly, 5 ng/μL of Dsg2 (Leinco, Fenton, USA) diluted in a carbonate-bicarbonate solution (pH 7.4) were incubated (50 μL/well) in a 96-well microplate (Sigma-Aldrich, St. Louis, USA) at 4 °C overnight. After four washes with 0.05% PBS/Tween and incubation with a blocking solution (PBS/Tween 0.05%/5% skim milk) for one hour (h), 50 μL of serum samples (diluted to 1: 100 in the blocking solution), in duplicate, were added and incubated at room temperature (RT) for 2 hours. After four washes, 50 μL (diluted to 1:5000 in the blocking solution) of HRP antibody conjugated to anti-human IgG (Abcam, Cambridge, USA) were incubated at RT for 2 hours. After four new washes, 50 μL of ABTS peroxidase substrate (2,2ʹ-azino-bis(3-ethylbenzothiazoline-6-sulfonic acid)) were added and kept at RT for 15 minutes (min) in the dark, and the reaction was finally stopped with 50 µL of 1 M H_2_SO_4_. The optical density (OD) titers were determined using an ELISA plate reader (Biochrom Asys Expert Plus Microplate Reader, Cambourne, UK) at 405 nm. Commercial anti-Dsg2 antibody (Abcam, Cambridge, USA) was used as a positive control. A cut-off value of 0.1365 OD was established by the ROC curve (Supplemental Fig. 1). Titers > 0.400 OD were obtained in four samples from the CTL group and were excluded from the calculation because they exceeded the normality curve.

### Adsorption ELISA for anti-Dsg2 specificity

To exclude a cross-reaction of anti-Dsg2 antibodies with Dsg1 and Dsg3 proteins, six serum samples from the PF and six from the PV groups, with high titers of anti-Dsg 1, 2 and 3 were selected. Serum adsorption ELISA assays were performed with Dsg2 (Leinco, Fenton, USA) until the anti-Dsg2 level was below the cut-off value. Subsequently, the remaining serum was placed in commercial anti-Dsg1 and anti-Dsg3 ELISA plates (MBL, Nagoya, Japan) to compare pre-and post-adsorption titers.

### Biopsy samples

Skin and/or mucosal biopsies were performed in three patients with PF and three with PV, who were treatment-naïve or who had active lesions without treatment for more than 60 days. Biopsies were performed in intact perilesional skin (IS) and bullous lesions, named as lesional skin (LS) or lesional mucosa (LM). As a control, an intact mucosal (IM) biopsy was taken from a patient with PF. An abdominoplasty skin sample and a PF IM sample were used as controls in the IHC analysis, and two facelift (FL) surgery skin samples were used as controls in the qPCR assay. Four-mm punch biopsies were divided into two fragments: one was stored in Tissue-Tek medium (Sakura Finetek, Torrance, USA) for staining with hematoxylin-eosin and immunohistochemistry (IHC), and the other was stored in RNAlater® solution (Life Technologies, Carlsbad, USA) at −80 °C for RNA extraction and subsequent qPCR assay.

### Immunohistochemistry (IHC)

The HRP-Polymer MACH 1 Detection System (Biocare Medical, Concord, USA) was used according to the manufacturer’s recommendations. Frozen fragments were fixed in acetone (Merck, Darmstadt, Germany) at 4 °C for 10 minutes and washed in PBS pH 7.4; the endogenous peroxidase activity was inhibited by incubation with 0.3% H_2_O_2_ (Labsynth, Diadema, Brasil) for 10 minutes. Then, the fragments were incubated with Sniper blocking solution at RT for 10 minutes, followed by the addition of 5 µg/mL of anti-Dsg1, anti-Dsg2, and anti-Dsg3 monoclonal antibody (Abcam, Cambridge, UK) for 2 hours. The sections were washed with PBS for 3 minutes twice. Then, the probe was added for 15 minutes. The sections were washed in PBS again and incubated with the HRP polymer for 30 minutes. After the washing, the reaction was completed by adding the chromogenic substrate diaminobenzidine for 2 minutes, prepared according to the manufacturer’s instructions (Biocare Medical, Concord, USA). The sections were then contrasted with Harris’ hematoxylin (Merck Millipore, Burlington, USA) for 40 seconds and washed in running water. To mount the slides, the sections were dehydrated in ethanol baths with increasing concentrations and diaphanized in a xylene bath and then mounted with Entellan (Merck, Darmstadt, Germany), and the sections were coverslipped for analysis under optical microscopy (Imager- A1, Zeiss, Germany).

### RNA extraction and cDNA synthesis

Total RNA was extracted from skin or mucosal biopsy samples using Trizol reagent, according to the manufacturer’s instructions (Invitrogen, Carlsbad, USA). After extraction, the samples were incubated with DNAse (Promega, Madison, USA) and quantified in a NanoVue® Plus spectrophotometer (GE Healthcare Life Sciences, Chicago, USA). A 40 ng RNA sample was reversely transcribed with the cDNA synthesis kit (Promega, Madison, USA) according to the manufacturer’s instructions.

### Real-time quantitative polymerase chain reaction (qPCR) and sequencing of Dsg1, Dsg2 and Dsg3

SYBR Green qPCR was performed in a final volume of 22.5 μL, with 12.5 μL of SYBR Green PCR MIX (Promega, Madison, USA), 0.5 μL of each primer (10 μM), 6.5 μL of ultrapure water and 2.5 μL of cDNA (1:4) in the Rotor Gene-Q® thermocycler (Qiagen, Hilden, Germany). Primer sequences that amplify fragments of the Dsg1, Dsg2 and Dsg3 genes and of the human ribosomal gene (18S) as endogenous genes were used.^13^, ^14^, ^15^ The primer sequences, the resulting amplified product (amplicon) and the amplification cycle parameters are detailed in Supplemental Table 1. The relative gene expressions of Dsg1, Dsg2 and Dsg3 were calculated using the equation 2 ^(−ΔcT)^, having the 18S rRNA as the reference gene; ΔcT was calculated by subtracting the cycle threshold (cT) value from the target gene (Dsg1, Dsg2 or Dsg3) from the cT value of the reference gene (18S).^16^ To verify the specificity of each sequence, the obtained amplicon was purified (ExoSAP-IT®), sequenced (3500 Genetic Analyzer, Applied Biosystems, USA), and analyzed at the NCBI BLAST.

### Statistical analysis

Categorical variables were analyzed using the chi-square test (×2) or Fisher’s exact test when data were scarce. Kruskall-Wallis test, followed by Dunn’s test, was used to compare ELISA titers between the groups. Spearman’s test was used to correlate antibody titers against Dsgs, as well as with disease duration and patient age. Friedman’s test was used to compare serial pre- and post-serum adsorption anti-Dsg2 titers. Wilcoxon’s test was applied to compare anti-Dsg1 and anti-Dsg3 titers pre- and post-adsorption with Dsg2. Multivariate logistic regression analysis with SPSS Statistics 17.0 (IBM SPSS® software, USA) was used to confirm the significant results obtained in the univariate analysis. Significance was set at α = 5%. The GraphPad Prism 8.1 software was used for statistical analysis and for the construction of graphs (GraphPad software Inc., La Jolla, USA).

## Results

**Figure 1:**
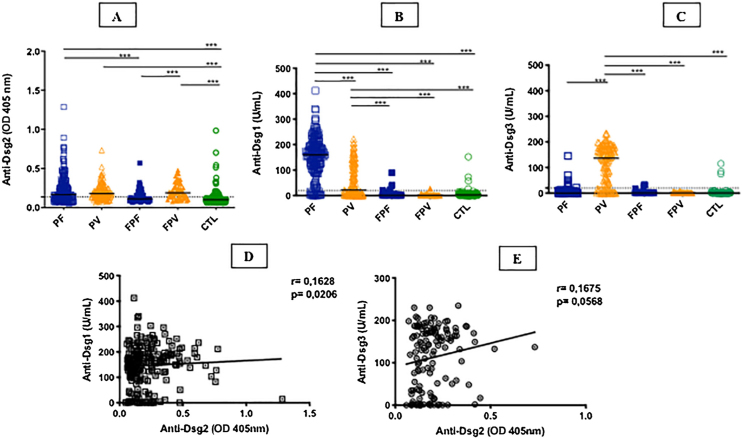
IgG antibodies against Dsg1, Dsg2 and Dsg3 in the studied groups. **A**, Anti-Dsg2 by in-house ELISA. PF, PV and FPV had higher titers compared to controls (CTL; p < 0.0001); PF and FPV showed higher titers than FPF (p = 0.0046 and p = 0.0004, respectively). **B**, Anti-Dsg1 by commercial ELISA. PF had higher titers compared to all groups (p < 0.0001); PV, higher titers than FPF, FPV and CTL (p < 0.0001). **C**, Anti-Dsg3 by commercial ELISA. PV had higher titers than all groups (p < 0.0001). The horizontal line in each column indicates the median; the dotted line, the cut-off value. PF, Pemphigus Foliaceus; PV, Pemphigus Vulgaris; FPF, Family members of PF, FPV, Family members of PV. Statistical analysis: Kruskal-Wallis followed by Dunn’s test. **D**, Spearman’s correlation between anti-Dsg2 and anti-Dsg1 titers in PF. **E**, Spearman’s correlation between anti-Dsg2 and anti-Dsg3 titers in PV.

Table 1 summarizes the demographic, clinical, and laboratory data of the studied groups. The PF and PV groups had similar and higher anti-Dsg2 titers (OD) when compared to the CTL group (0.166; 0.180; 0.102, respectively; p < 0.0001; Fig. 1A). Also, the PF group had higher anti-Dsg2 titers compared to the FPF (p = 0.0046) and FPV (p = 0.0004) groups. These results remained statistically significant after the multivariate analysis, corrected for age, sex, and antibody titers against Dsg1 and Dsg3. The production of autoantibodies against Dsg1 and/or Dsg3 was observed in the groups of family members and CTL (Fig. 1B–C). Anti-Dsg2 titers were higher in the treatment-naïve PF and PV groups when compared to the CTL group (p < 0.0001), and in the untreated PV group when compared to the PV group undergoing treatment (p = 0.0311; Supplemental Fig. 2), remaining significant after the multivariate analysis (p = 0.0500). The PF subgroups did not differ regarding the anti-Dsg2 titers (data not shown).

The seroprevalence of anti-Dsg2 in the PF, PV and FPV groups was similar (63.9%; 66.4%; 73.7%, respectively) and higher than in the FPF and CTL groups (38% and 27.2%, respectively; p < 0.0001; Table 1).

There was no significant correlation of anti-Dsg2 titers with disease duration in PF (p = 0.5365) and PV (r = -0.1629; p = 0.0630; data not shown), as well as with the clinical form of PF (p = 0.9636) and PV (p = 0.3909; Table 1). There was no significant difference in the anti-Dsg2 titers in the PF and PV subgroups with active skin lesions and without skin lesions (p = 0.3790 and p = 0.1312, respectively; data not shown).

There was a positive correlation of anti-Dsg2 titers with anti-Dsg1 titers in the PF group (r = 0.1628; p = 0.0206), with a trend towards a positive correlation with anti-Dsg3 titers in PV (r = 0.1675, p = 0.0568; Fig. 1D–E). Considering the treatment-naïve subgroups, and the untreated for more than 60 days and with active lesions subgroups, there was no correlation between anti-Dsg2 titers and anti-Dsg1 titers in PF (r = 0.2100, p = 0.0650), as well as with the anti-Dsg3 titers in PV (r = 0.1481, p = 0.4264; data not shown).

After adsorption of sera from patients with PF and PV with the Dsg2 peptide, anti-Dsg1 and anti-Dsg3 titers remained similar to the pre-adsorption ones (p > 0.9999 and p = 0.4063, respectively), confirming the specificity of anti-Dsg2 antibodies (Supplemental Tables 2 and 3).

### Protein expression of desmogleins by IHC

Table 2 shows the demographic and serological data of patients with PF and PV submitted to skin and/or mucosa biopsy for IHC analysis.

**Table 2:** Demographic, clinical and anti-Dsg 1, 2 and 3 antibody titers by ELISA of patients with PF and PV submitted to skin and/or mucosa biopsy.

	Age	Sex	Disease duration (months)	Clinical form	Treatment on biopsy date	ANTI-DSG1 (U/ML)^a^	ANTI-DSG2 (OD)^b^	ANTI-DSG3 (U/ML)^a^
**PF1**	23	Male	48	Generalized	In treatment for 30 days	99.80	0.088	1.93
**PF2**	18	Male	6	Generalized	Treatment naïve	193.08	0.223	4.15
**PF3**	25	Male	36	Localized	Treatment naïve	237.90	0.894	8.70
**PV1**	61	Male	24	Muco-cutaneous	No treatment for more than 60 days	3.87	0.211	172.78
**PV2**	50	Male	4	Muco-cutaneous	Treatment naïve	42.16	0.213	166.91
**PV3**	68	Male	6	Muco-cutaneous	Treatment naïve	167.62	0.311	163.80

a Anti-Dsg1 and 3 cut-off = < 20 U/mL (ELISA, MBL, Japan).

b Anti-Dsg2 cut-off = 0.1365 DO (in-house ELISA).

**Figure 2:**
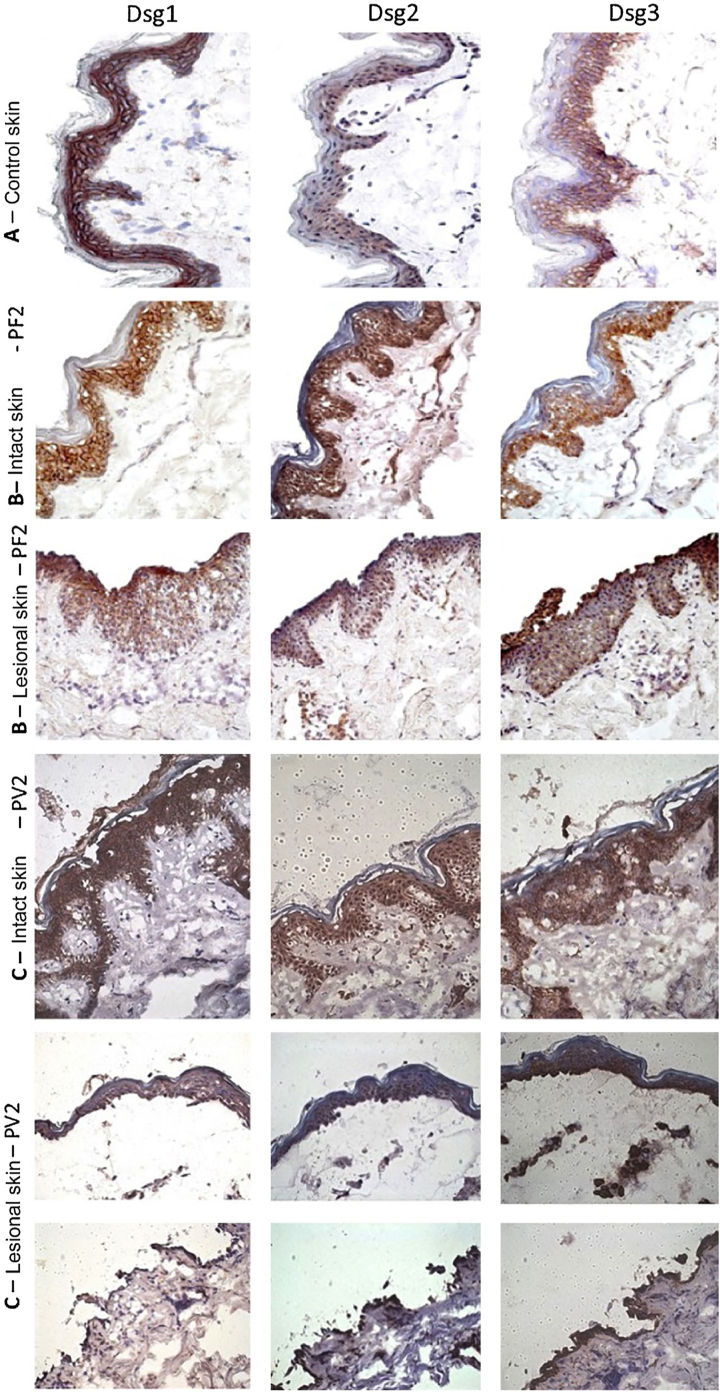
Immunohistochemistry panel with Dsg1, Dsg2 and Dsg3 expressions in skin samples (original magnification: ×40). (A), Abdominoplasty control skin sample showing typical distribution of Dsg1, Dsg2 and Dsg3 in the epidermis. (B), PF - intact skin sample showing increased expressions of Dsg2 and Dsg3 in the entire epidermis and PF - lesional skin sample showing increased expressions of Dsg2 and Dsg3 on the roof and floor of the acantholytic bulla. (C), PV-intact skin sample showing increased expression of Dsg2 and Dsg3 in the entire epidermis and PV-lesional skin sample showing increased expression of Dsg2 and Dsg3 on the roof and floor of the bulla and in the acantholytic cells.

The classic epidermal distribution of Dsgs was confirmed in the abdominoplasty control skin sample: Dsg1 was expressed throughout the epidermis, predominantly in the upper layers, while Dsg2 and Dsg3 predominated in the lower layers. The expression of Dsg2 was less intense than that of Dsg1 and Dsg3 (Fig. 2A). Different from the control skin sample, Dsg2 expression was more intense in all layers of the epidermis in IS samples from the PF and PV groups (Fig. 2B–C). The LS samples of the PF group showed intense expression of Dsg2 on the floor of the bulla and, in the PV group, on the roof and floor of the bulla, including its expression in acantholytic cells (Fig. 2B–C).

**Figure 3:**
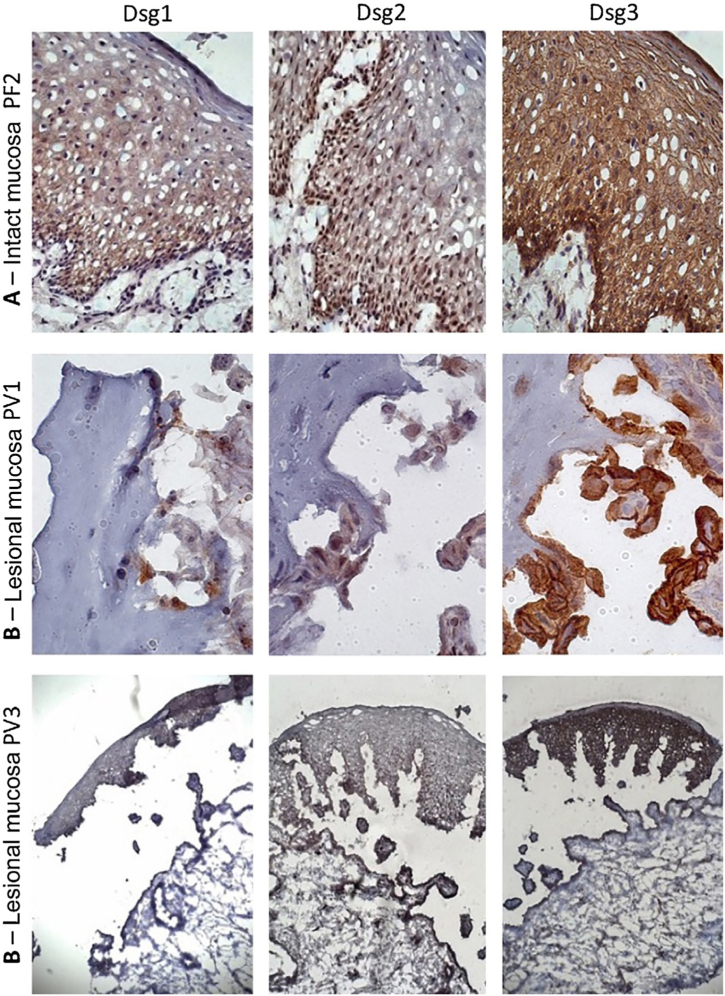
Immunohistochemistry panel with Dsg1, Dsg2 and Dsg3 expressions in mucosal samples. (A), PF-intact mucosa showing the typical distribution of Dsg1, Dsg2 and Dsg3 in the epithelium (original magnification: ×63). (B), PV-lesional mucosa showing Dsg2 and Dsg3 expression surrounding the acantholytic bulla (original amplification: ×100 and ×40).

Similar to the control skin sample, Dsg2 expression in the PF IM sample was more intense in the lower layers. Moreover, there was a predominance of Dsg3 expression in all layers of the epidermis, compared to those of Dsg1 and Dsg2 (Fig. 3A). In the LM of the PV group, there was a predominance of Dsg3 expression on the roof and floor of the bulla (Fig. 3B–C).

**Figure 4:**
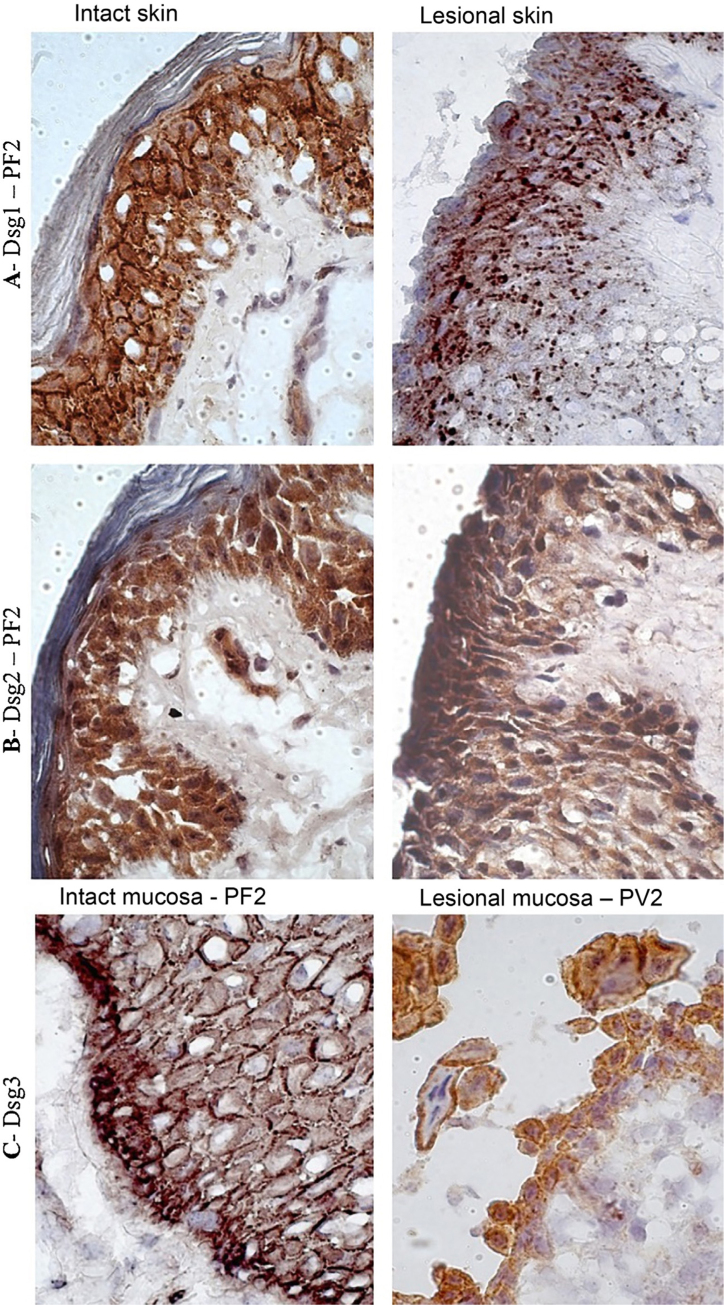
Immunohistochemistry panel with Dsg1, Dsg2 and Dsg3 expressions in skin and mucosa samples from patients with PF and PV. (A), Dsg1 in intact and lesional skin samples from patient PF2 showing conspicuous intercellular and intracytoplasmic granules (Dsg1 internalization), widely distributed in lesional skin (original magnification: ×100). (B), Dsg2 comparing intact and lesional skin sample from patient PF2, confirming the absence of granules (original magnification: ×100). (C), Dsg3 comparing intact mucosa sample from patient PF2 and lesional mucosa from patient PV3, showing fine intercellular and intracytoplasmic granules (Dsg3 internalization) in the lesional mucosa (original magnification: ×100).

In the PF LS sample, conspicuous intercellular and intracytoplasmic granules corresponding to the internalization of Dsg1, were observed along the entire length of the bulla floor, from the basal layer to the Malpighian layer (Fig. 4A). Intracytoplasmic granules, which could represent the internalization of Dsg2, were not observed in the lesion samples of the PF and PV groups (Fig. 4B). Fine and sparse intercellular granules of Dsg3 were demonstrated in the LS and LM samples of the PV group (Fig. 4C).

### Gene expression of desmogleins by qPCR

**Figure 5:**
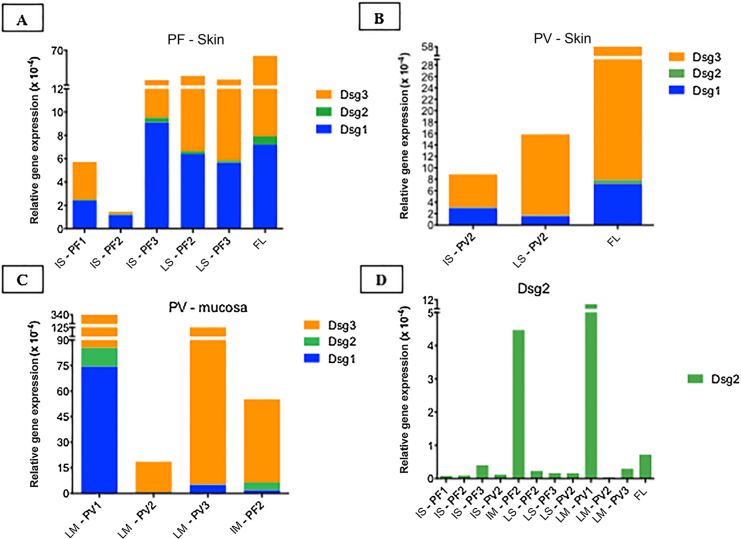
Relative gene expressions (×10-4) of Dsg1, Dsg2 and Dsg3 in skin and mucosal samples. (A), Intact skin (IS) lesional skin (LS) samples of the PF group compared to the facelift (FL) control sample showing predominance of Dsg3 expression in comparison to Dsg1 and Dsg2. (B), IS and LS samples of the PV and FL groups. The expressions of Dsg1, Dsg2 and Dsg3 were lower than in the FL sample. (C), Lesional mucosa (LM) sample of the PV group in comparison to intact mucosa (IM) sample of the PF group showing predominance of Dsg3 expression in comparison to Dsg1 and Dsg2. Dsg2 expression is increased in mucosal samples. (D), Comparison of Dsg2 expression in all PF and PV samples. Relative gene expressions of Dsg1, Dsg2, Dsg3 were calculated using 2^(−ΔcT)^ and using 18S rRNA as the reference gene.

Table 2 shows the demographic and serological data of patients with PF and PV submitted to skin and/or mucosa biopsy for qPCR assays. The amplification curves of the cDNA samples are shown in Supplemental Fig. 3. The results of the relative gene expressions of Dsg 1, 2 and 3 are shown in Fig. 5.

Considering the control FL skin sample, there was a predominance of relative gene expression of Dsg3, followed by expressions of Dsg1 and Dsg2 (Fig. 5A–B). The relative gene expressions of Dsg1 and Dsg3 were lower in the PV, followed by the PF group, with a similar distribution of Dsgs to the FL sample (Fig. 5A–B).

The expressions of Dsg 1, 2 and 3 showed different results in the IM samples from the PF group and LM from the PV group, with a predominance of Dsg3 expression (Fig. 5C). The expression of Dsg2 was shown to be higher in the IM sample of the PF and LM of the PV group, compared to the IS and LS samples of the PF and PV groups (Fig. 5D).

## Discussion

In the present study, we demonstrated the production of serum IgG autoantibodies against Dsg2 in a large sample of patients with PF and PV, compared to the FPF, FPV and CTL groups, all inhabitants of a region prevalent in PF and PV in Southeastern Brazil.^3^ The seroprevalence of anti-Dsg2 in the PF group (63% of 202 PF) was higher when compared to the study by Flores et al. (2012; 40% of 106 PF), which was carried out in an endemic region for PF in Brazil.^17^ The seroprevalence in the CTL group was similar in both (20% and 27%, respectively).

Due to the protein similarity of Dsg1, Dsg2 and Dsg3, a possible cross-reaction of anti-Dsg2 antibodies with Dsg1 and Dsg3 proteins was suggested.^5^ However, adsorption tests did not show differences in anti-Dsg1 and anti-Dsg3 titers when the pre- and post-adsorption results were compared with Dsg2, confirming the specific anti-Dsg2 production.

Considering the specificity of the production of autoantibodies against Dsg2, some questions must be considered: (a) Would they be pathogenic? and (b) Would they be produced by the epitope-spreading phenomenon?^18^

As for the first question, the analysis of the results points to the non-pathogenicity of anti-Dsg2 antibodies in pemphigus. Higher anti-Dsg2 titers were detected in the PF, PV and FPV groups when compared to the FPF and CTL groups. The production of anti-Dsg1 and anti-Dsg3 by healthy individuals is known; therefore, the presence of anti-Dsg2 in the FPF, FPV, and CTL groups would not exclude its participation in the pathogenesis of PF and PV.^19^, ^20^, ^21^ Anti-Dsg2 titers correlated with those of anti-Dsg1 in PF (r = 0.1628; p = 0.0206), also reported by Flores et al. (2012);^17^ however, there was no correlation of anti-Dsg2 with anti-Dsg3 in PV. Moreover, anti-Dsg2 titers did not correlate with the clinical form of disease activity in the PF and PV groups, in contrast to anti-Dsg1 and anti-Dsg3 serum titers, which are associated with PF and PV severity.^22^, ^23^ Additionally, higher anti-Dsg2 titers were found in the untreated PF and PV groups when compared to the CTL group, and in the untreated PV group when compared to the treated PV group. Thus, the decrease in anti-Dsg2 after immunosuppressive treatment could favor the pathogenicity of anti-Dsg2 in PV; however, this result was not confirmed in the PF group. Concluding this discussion, the expression of Dsg2 in tissues other than the skin is known, such as lung, gastrointestinal, renal, and myocardial tissues.^5^, ^8^ Thus, corroborating that anti-Dsg2 antibodies are not pathogenic, there is no evidence of involvement of internal organs in pemphigus, with the exception of the esophagus in the PV group.^24^

As for the second question, it would be more plausible to consider that anti-Dsg2 antibodies were produced by the epitope-spreading phenomenon in PV since Dsg2 and Dsg3 are more expressed in the lower layers of the epidermis. Moreover, patients with PV had higher anti-Dsg2 titers prior to the start of the immunosuppressive treatment. Hence, one could suppose that Dsg2 is exposed at the beginning of the acantholytic process, produced by anti-Dsg3, with the consequent production of anti-Dsg2 by the epitope-spreading phenomenon.^18^ However, similar anti-Dsg2 titers were observed in PF and PV. Thus, the epitope-spreading phenomenon would be occurring in both PF and PV pathogenic processes. This discussion will be resumed after considering the IHC results.

Since patients with PF and PV produced anti-Dsg2, it was decided to verify the protein and gene expressions of Dsg2 *in situ* through IHC and qPCR, respectively, and compare the results with the expressions of Dsg1 and Dsg3. The IP and LS samples of the PF and PV groups were compared through IHC and qPCR in the abdominoplasty and FL skin samples, respectively. The LM of the PV group was compared to the IM of the PF group.

In the abdominoplasty control sample, the protein expression of Dsgs confirmed the classic distribution pattern in epidermal layers, with the expression of Dsg2 being restricted to the lower layers. Also, Dsg1 and Dsg3 were predominantly expressed in keratinocyte membranes.^5^, ^12^ Interestingly, the expressions of Dsg2 and Dsg3 in IS samples from the PF and PV groups were more intense than in the abdominoplasty skin sample and extended to all layers of the epidermis. In the LS of PF and PV groups, Dsg2 expression was intense on the roof and floor of the bulla and in acantholytic cells. Moreover, in LS samples from the PF and PV groups, the presence of intra- and intercellular Dsg1 and Dsg3 granules was observed in keratinocytes, respectively. Dsg1 granules extended through the entire Malpighian layer over the floor of the PF acantholytic bulla, confirming the internalization of Dsg1 described thirty years ago.^25^, ^26^, ^27^, ^28^ The internalization of Dsg3 in the LS sample of the PV group was less evident but has been commonly described.^29^, ^30^, ^31^, ^32^ While Dsg2 was overexpressed in IS, its internalization was absent in LS samples of the PF and PV groups, representing further evidence that anti-Dsg2 antibodies have no pathogenic role in pemphigus acantholytic process.

In the FL skin sample, the relative gene expression of Dsg2 was lower than that of Dsg1 and Dsg3, consistent with the protein expression evaluated by IHC. Surprisingly, the relative gene expression of Dsg2 was increased in the IM of the PF and LM of the PV group.

The role of protein and gene overexpression of Dsg2 in skin/mucosal samples from patients with PF and PV deserves attention. Iwatsuki et al. (1999) showed that the internalization of Dsg1 and Dsg3 preceded acantholysis in skin samples from PF and PV and was associated with the induction of Dsg2 expression to compensate for the depletion of Dsg1 and Dsg3.^13^ Moreover, Moll et al. (1999) demonstrated a strong expression of Dsg2 in a skin culture model created to study epithelial healing.^33^ In transgenic mice overexpressing Dsg2, Brennan et al. (2010) demonstrated the hyperproliferation of epidermal keratinocytes and decreased apoptosis and showed that the overexpression of Dsg2 restricted the formation of epidermal bullae by IgPF.^34^, ^35^ Subsequently, Hartlieb et al. (2014) demonstrated that although Dsg2 contributes less than Dsg3 to keratinocyte cohesion, Dsg2 compensated for Dsg3 loss of function.^6^, ^36^ More recently, Sigmund et al. (2020) demonstrated that Dsg2 shows heterophilic binding to Dsg3 and can minimize the loss of adhesion between keratinocytes in the acantholytic process.^37^

Adding our results to the reports in the literature, Dsg2 overexpression in the skin and mucosa of the PF and PV groups seems to be in line with the aforementioned reports: the overexpression of Dsg2, to compensate for the loss of function of Dsg1 and Dsg3, might afford protection against acantholysis and be involved in tissue repair. Moreover, the overexpression of Dsg2 in all layers of the epidermis in the IS and LS of the PF and PV groups might justify the production of autoantibodies against Dsg2 through the epitope-spreading phenomenon.

Finally, although anti-Dsg1 and anti-Dsg3 autoantibodies are reported in normal populations, the results of increased anti-Dsg2 titers in the FPV group should be further evaluated.^19^, ^20^, ^21^

## Study limitations

The present study had a small sample size for the statistical analysis of the protein and gene expressions of Dsg2 in the tissues.

## Conclusion

The present results allow the conclusion that autoantibodies against Dsg2 are not pathogenic in pemphigus and that protein and gene overexpression of Dsg2 in the skin and mucosal samples from patients with PF and PV may be involved in the protection against acantholysis.

## Submission and verification statement

The partial results of the ELISA test were presented as a poster entitled “Antibodies against desmoglein 2 are present in samples from patients with pemphigus foliaceus and pemphigus vulgaris and in family members and controls from an endemic region” at the XXXIV Annual Meeting of Latin American Dermatologists, 2016, São Paulo, having been awarded the third place.

## Financial support

The study was partially funded by 10.13039/501100001807FAPESP (*Fundação de Amparo à Pesquisa do Estado de São Paulo*) (#2010/51729-2) and FAEPA (*Fundação de Apoio ao Ensino, Assistência e Pesquisa*). TJ received a PhD scholarship from FAPESP (#2016/09011-3) and SV received a PhD scholarship from CAPES (*Coordenação de Aperfeiçoamento de Pessoal de Nível Superior*).

## Authors’ contributions

Marcela Calixto Brandão Miguel: Contributed with the collection, analysis and interpretation of data; drafting and editing of the manuscript; statistical analysis; critical review of the literature.

Tamiris Amanda Julio: Contributed with the collection, analysis and interpretation of data; critical review of the manuscript; approval of the final version of the manuscript.

Sebastian Vernal: Contributed with the data analysis; critical review of the manuscript; approval of the final version of the manuscript.

Natália Aparecida de Paula: Contributed with the collection, analysis and interpretation of data; critical review of the manuscript; approval of the final version of the manuscript.

Andre Lieber: Contributed with the planning of the study, critical review of the manuscript; approval of the final version of the manuscript.

Ana Maria Roselino: Contributed with the design and planning of the study; analysis and interpretation of data; critical review of the literature; critical review of the manuscript and effective participation in research orientation.

## Conflicts of interest

None declared.
