# Supplementary material for: Autoantibodies against desmoglein 2 are not pathogenic in pemphigus
Source: An Bras Dermatol. 2022 Jan 17;97(2):145–56. doi: 10.1016/j.abd.2021.06.004 (PMC9073259; doi:10.1016/j.abd.2021.06.004)
Supplement: Supplementary file 1 [file mmc1.doc]

**JPED-D-21-00036 – Supplementary Material**

**Supplemental Table 1** Forward (F) and Reverse (R) primers and qPCR cycling for Dsg 1, 2 and 3, and endogenous human ribosomal gene (18S).

| **Target gene** | **Amplicon size** | | **Sequence of primers** | **Cycling** | **References^a^** |
| --- | --- | --- | --- | --- | --- |
| Dsg1 | 411bp | F | 5' TCAAGTTCGCAGCAGCCTGT3' | 95°C for 2 min; 45 cycles: 94°C for 1 min; 57°C for 2 min; 72°C for 2 min | (13) |
|  |  | R | 5’ TGTTCGGTTCATCTGCGTCA 3’ |  |  |
| Dsg2 | 793bp | F | 5' CAGTAGCTTCCCAGTTCC3' | 95°C for 2 min; 45 cycles: 94°C for 1 min; 57°C for 2 min; 72^o^C for 2 min | (13) |
|  |  | R | 5'CTGTAAGCTTCATGAAAAATCAG 3' |  |  |
| Dsg3 | 107bp | F | 5' TGATCTGTCCCATTTCCAGTGT3' | 95°C for 2 min; 45 cycles: 95°C for 15 sec; 60°C for 60 sec | (14) |
|  |  | R | 5’ TCATATTAGACGGGAGCAAGGA3’ |  |  |
| 18S | 151bp | F | 5' GTAACCCGTTGAACCCCATT 3' | 95°C for 2 min; 45 cycles: 95°C for 15 sec; 60°C for 60 sec | (15) |
|  |  | R | 5' CCATCCAATCGGTAGTAGCG 3' |  |  |

^a^ Primer sequences were compiled from the cited references.

**Supplemental Table 2** Sequence of anti-Dsg2 values in OD of sera from patients with PF and PV after four adsorption assays of sera with Dsg2 peptide by indirect ELISA (1^st^ to 4^th^ Assay).

|  | **Pre-** **adsorption ^a^** | **1^st^ assay** | **2^nd^ assay** | **3^rd^ assay** | **4^th^ assay** | **p** |
| --- | --- | --- | --- | --- | --- | --- |
| **PF1** | 0.358 | 0.232 | 0.183 | 0.140 | 0.062 | 0.0008 |
| **PF2** | 0.573 | 0.391 | 0.241 | 0.156 | 0.081 |  |
| **PF3** | 0.241 | 0.188 | 0.093 | 0.098 | 0.054 |  |
| **PF4** | 0.322 | 0.220 | 0.052 | 0.136 | 0.063 |  |
| **PF5** | 0.296 | 0.241 | 0.034 | 0.097 | 0.067 |  |
| **PF6** | 0.186 | 0.188 | 0.029 | 0.082 | 0.057 |  |
| **Median** | 0.309 | 0.226 | 0.073 | 0.117 | 0.063 |  |
| **PV1** | 0.670 | 0.165 | 0.144 | 0.131 | 0.069 | 0.0002 |
| **PV2** | 0.386 | 0.238 | 0.155 | 0.170 | 0.073 |  |
| **PV3** | 0.303 | 0.197 | 0.102 | 0.171 | 0.077 |  |
| **PV4** | 0.416 | 0.249 | 0.032 | 0.149 | 0.082 |  |
| **PV5** | 0.511 | 0.408 | 0.168 | 0.154 | 0.082 |  |
| **PV6** | 0.194 | 0.168 | 0.090 | 0.081 | 0.044 |  |
| **Median** | 0.401 | 0.218 | 0.123 | 0.152 | 0.075 |  |

^a^Anti-Dsg2 cut-off = 0.1365 OD (in-house indirect ELISA).

**Supplemental Table 3** Sequence of anti-Dsg1 and 3 autoantibody values pre- and post-serum adsorption with Dsg2 peptide determined by in-house indirect ELISA.

|  | **Pre-adsorption Anti-Dsg1 (U/mL)^a^** | **Post-adsorption Anti-Dsg1 (U/mL)** | **p** |
| --- | --- | --- | --- |
| **PF1** | 146.4 | 161.7 | 0.999 |
| **PF2** | 225.8 | 150.7 |  |
| **PF3** | 241.8 | 269.8 |  |
| **PF4** | 112.8 | 121.7 |  |
| **PF5** | 209.0 | 223.8 |  |
| **PF6** | 219.3 | 186.6 |  |
| **Median** | 214.2 | 174.2 |  |
|  | **Pre-adsorption Anti-Dsg3 (U/mL)^a^** | **Post-adsorption Anti-Dsg3 (U/mL)** |  |
| **PV1** | 185.9 | 185.2 | 0.406 |
| **PV2** | 158.5 | 181.5 |  |
| **PV3** | 150.5 | 175.4 |  |
| **PV4** | 152.5 | 151.8 |  |
| **PV5** | 169.9 | 155.7 |  |
| **PV6** | 137.1 | 174.0 |  |
| **Median** | 174.7 | 144.8 |  |

^a^Anti-Dsg1 and anti-Dsg3 cutoff = < 20 U/mL (ELISA, MBL, Japan).

**Supplemental  Figure 1** ROC curves with anti-Dsg2 (OD) ELISA values for patients with PF (**A**), PV (**B**) and PF + PV (**C**) in comparison to controls. The cut-off value was set at 0.1365 OD. PF, Pemphigus Foliaceus; PV, Pemphigus Vulgaris; AUC, Area Under the Curve


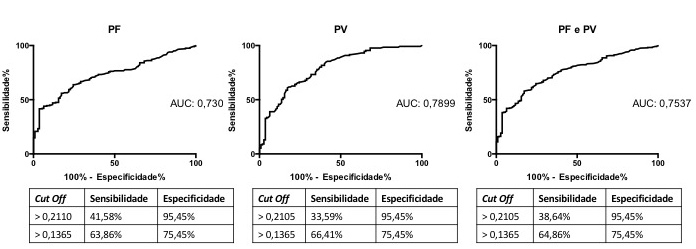


[REPLACE]

Sensibilidade = Sensitivity

Especificidade = Specificity

PF e PV = PF and PV

**Supplemental Figure 2** Anti-Dsg2 titers in patients with PV undergoing immunosuppressive treatment or not. Anti-Dsg2 titers were higher in the untreated group compared to those undergoing treatment (with or without active lesions; p = 0.0110 and p = 0.0311, respectively). The horizontal line in each group indicates the median; the dashed line, the cut-off value. Kruskal-Wallis test was used, followed by Dunn’s multiple comparison test.


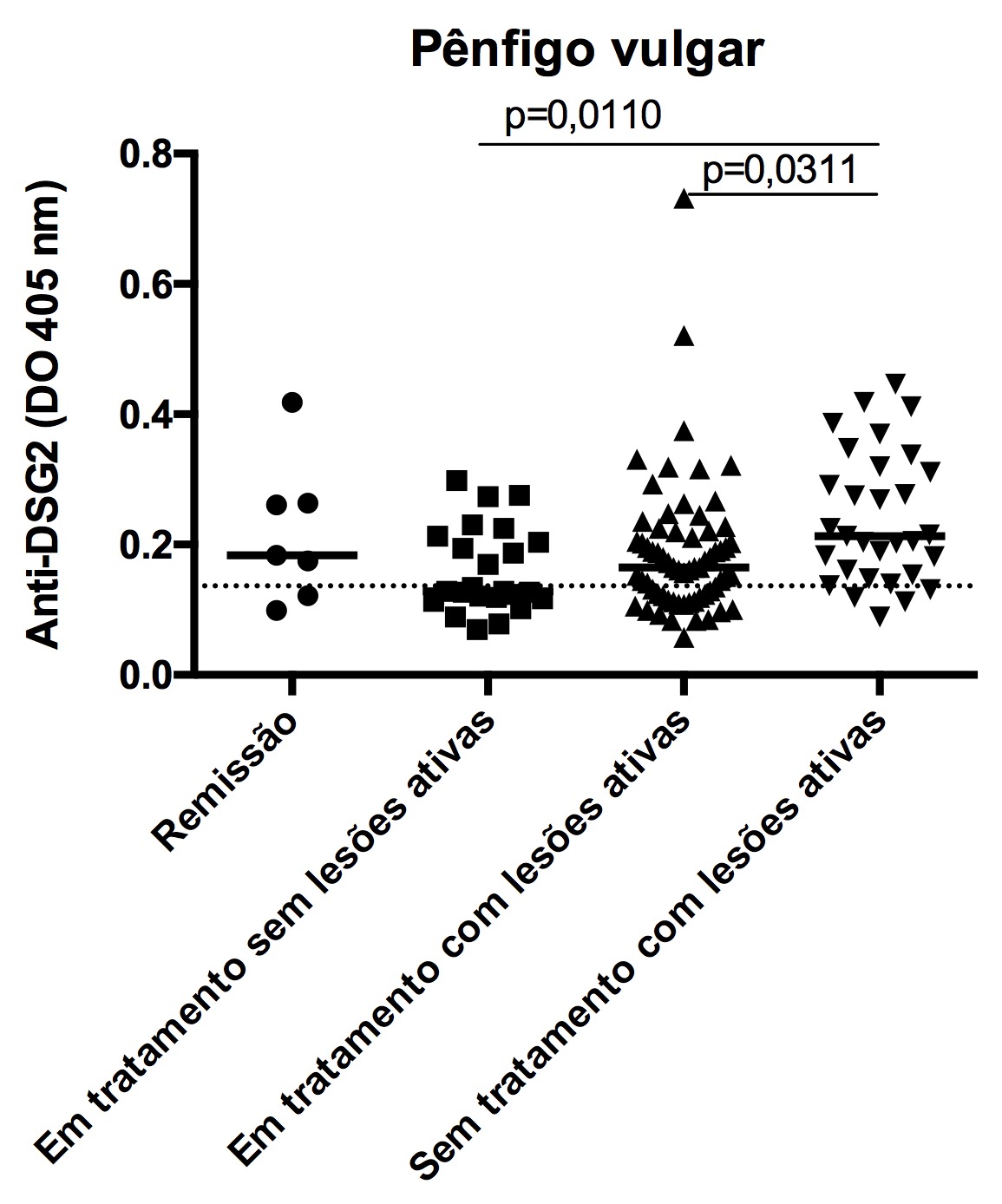


[REPLACE]

Pênfigo vulgar = Pemphigus vulgaris

DO = OD

Remissão = Remission

Em tratamento sem lesões ativas = Undergoing treatment, without active lesions

Em tratamento com lesões ativas = Undergoing treatment, with active lesions

Sem tratamento com lesões ativas = No treatment, with active lesions

**Supplemental Figure 3** cDNA amplification curves from the skin and/or mucosa samples from patients with PF and PV and facelift (FL) control for Dsg1, 2 and 3. The ordinate shows the emitted fluorescence, and the abscissa, the reaction cycles of the qPCR. The solid red horizontal line shows the threshold of the reaction. **A**, cDNA from samples for Dsg1 were amplified between 20 and 30 cycles. The two curves that precede the others correspond to the lesional mucosa samples from patient PV2. **B**, cDNA from samples for Dsg2 were amplified between 23 and 33 cycles. The four curves that precede the others correspond to the lesional mucosa samples from patient PV2 and the intact mucosa from patient PF2. **C**, cDNA from samples for Dsg3 were amplified between 18 and 32 cycles. The two curves that precede the others correspond to the lesional mucosa samples from patient PV2.

**
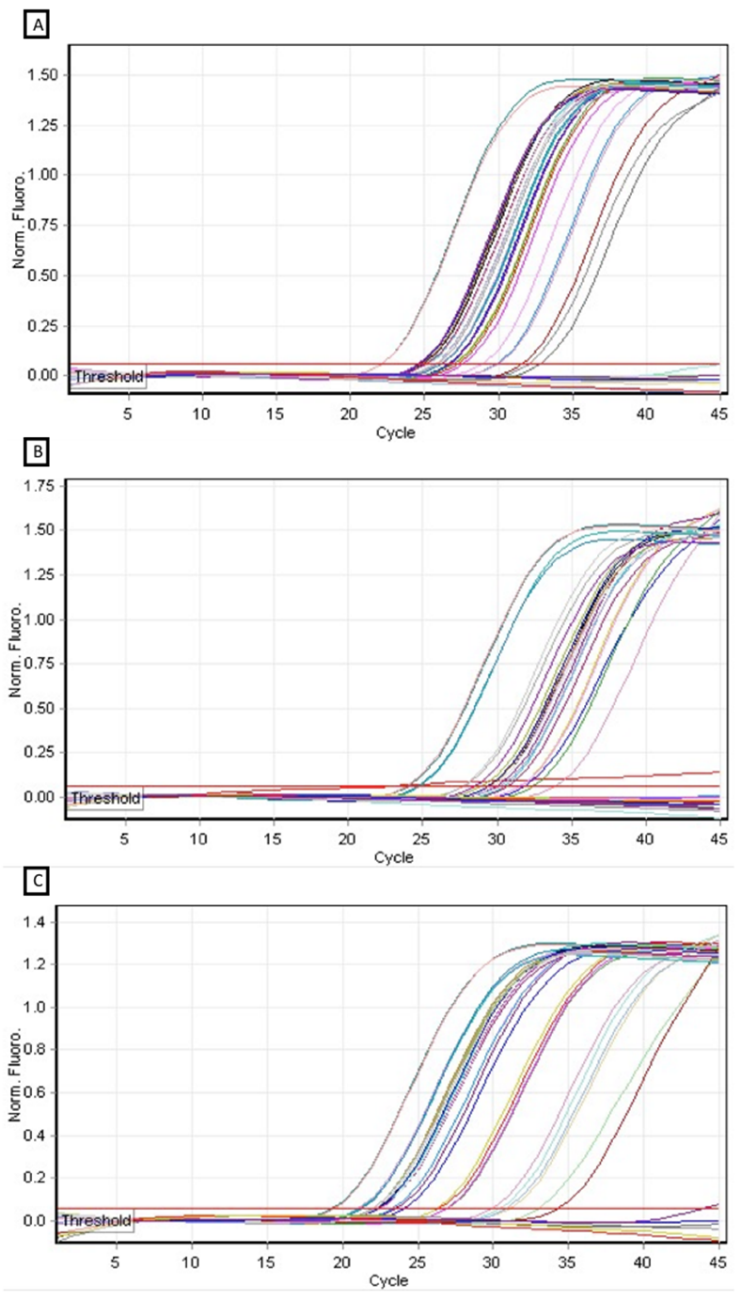
**

[REPLACE]

Cycle = Ciclo

Threshold = Limiar
